# Supplementary material for: Mechanistic insights into allosteric regulation of the A2A adenosine G protein-coupled receptor by physiological cations
Source: Nat Commun. 2018 Apr 10;9:1372. doi: 10.1038/s41467-018-03314-9 (PMC5893540; doi:10.1038/s41467-018-03314-9)
Supplement: Supplementary file 1 — Supplementary Information [file 41467_2018_3314_MOESM1_ESM.docx]

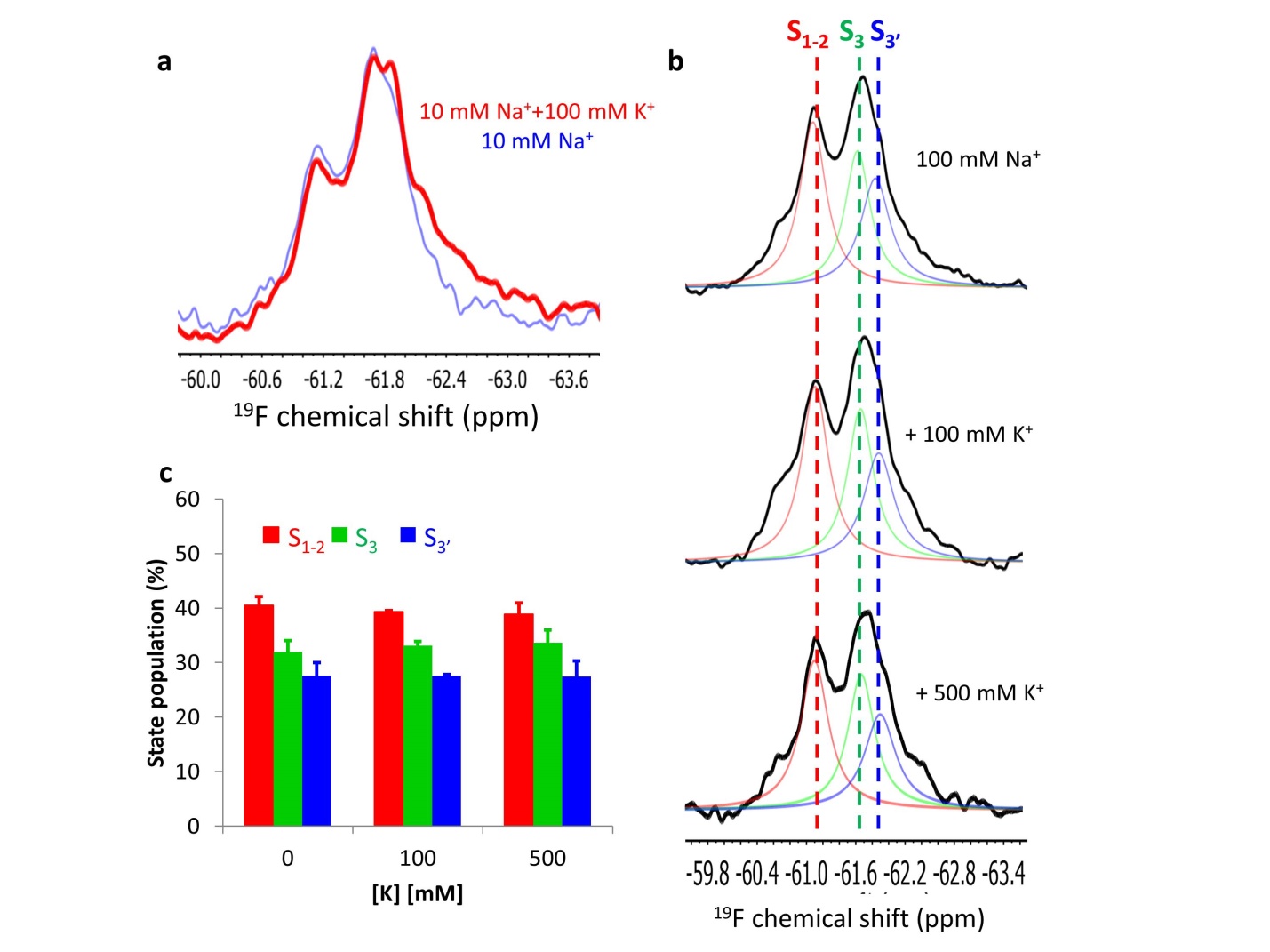


Supplementary Figure 1. Lack of effects of monovalent cations K^+^ on functional states of A_2A_R. a ^19^F NMR spectra of 200 μM BTFMA-labeled A_2A_R-V229C in 10 mM NaCl (blue) plus 100 mM KCl (red). b ^19^F NMR spectra of 100 μM BTFMA-labeled A_2A_R-V229C as a function of KCl concentration (0, 100, and 500 mM) in the presence of 100 mM NaCl. c Histogram of states, S_1-2_, S_3_ and S_3’_, upon addition of KCl with 100 μM receptor, in the presence of 100 mM Na^+^. Error-bars are defined based on the difference between the fitted area associated with the deconvolved spectrum and the area associated with the experimental spectrum. This error bar definition was applied to all spectra unless otherwise described.


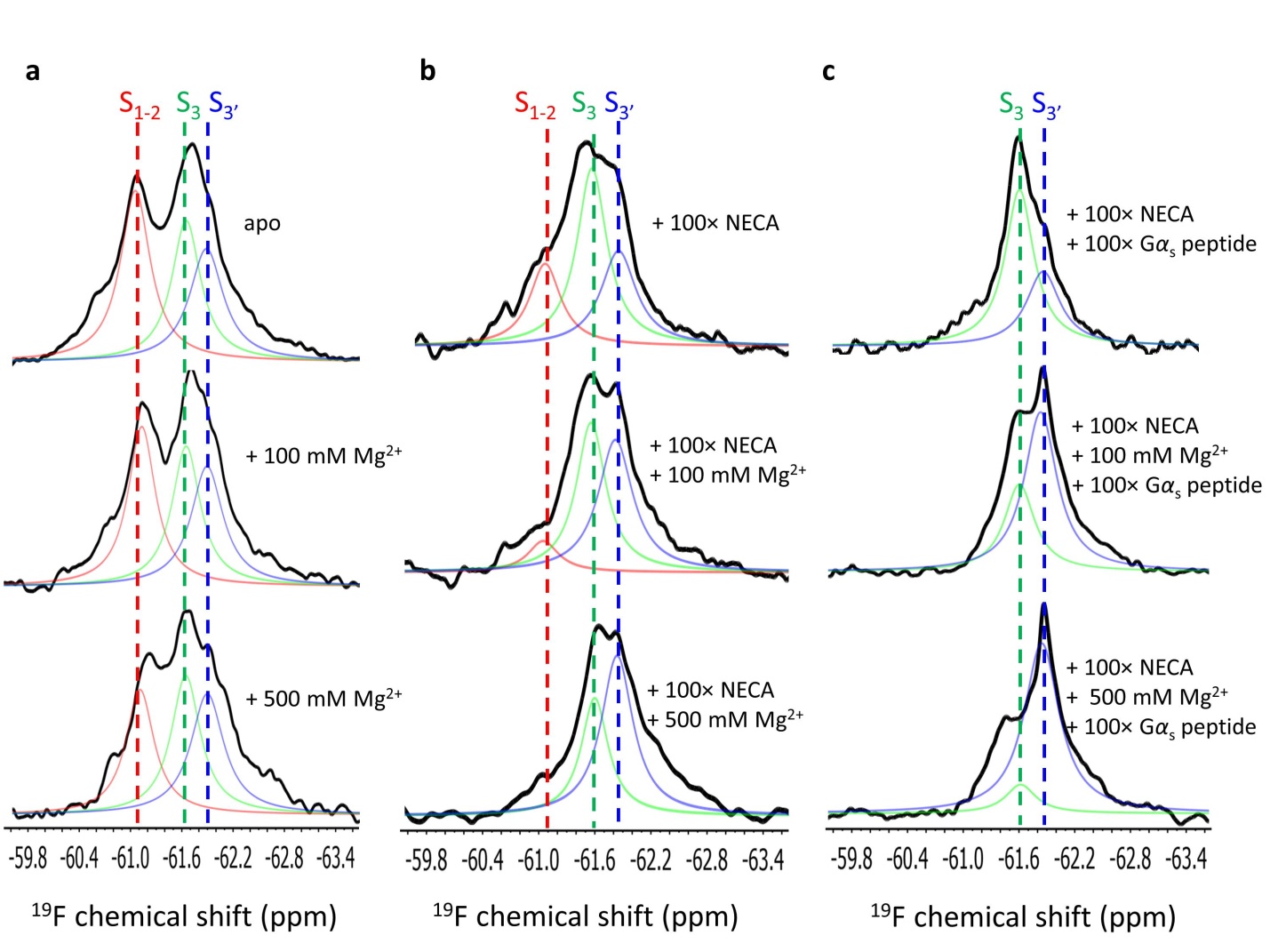


Supplementary Figure 2. Effects of Mg^2+^ on functional states of A_2A_R. All samples contain 100 mM NaCl. a ^19^F NMR spectra of 100 μM apo BTFMA-labeled A_2A_R-V229C as a function of Mg^2+^ concentration (0, 100 mM and 500 mM MgCl_2_). b ^19^F NMR spectra of 100 μM BTFMA-labeled A_2A_R-V229C in the presence of 100× excess NECA agonist as a function of Mg^2+^ concentration (0 mM, 100 mM and 500 mM MgCl_2_). c ^19^F NMR spectra of 100 μM BTFMA-labeled A_2A_R-V229C in the presence of 100× excess of both NECA and G*α*_s_ peptide as a function of Mg^2+^ concentration (0 mM, 100 mM and 500 mM MgCl_2_).


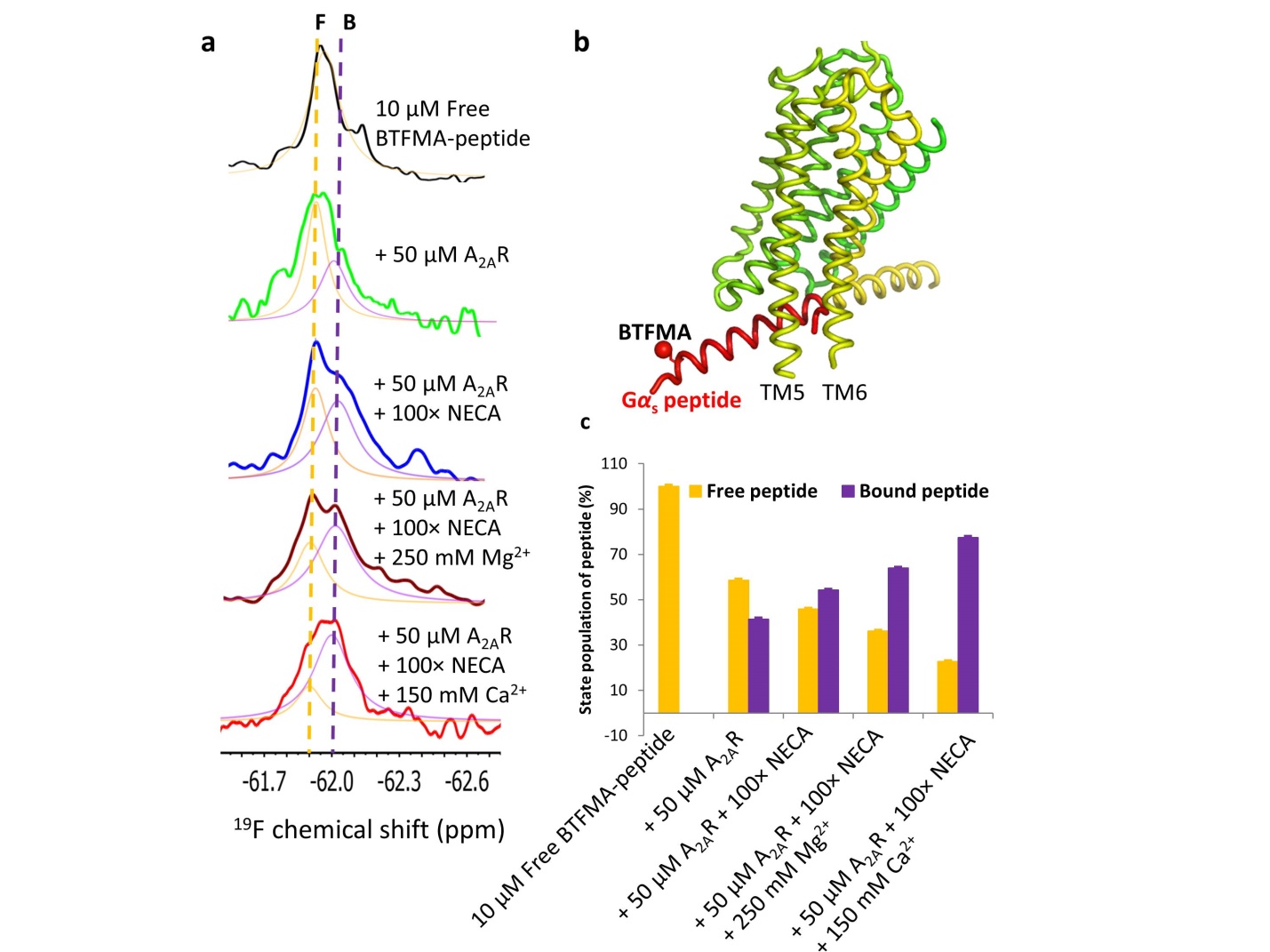


**Supplementary Figure 3. Effect of divalent cations on Gα_s_ peptide binding by ^19^F NMR**. **a** ^19^F NMR spectra of BTFMA-labeled Gα_s_ peptide (RVFNDCRDIIQRMHLRQYELL) as a function of NECA, Ca^2+^, and Mg^2+^. Deconvolutions of free and bound peptide states are represented in yellow and purple, respectively. **b** Model of G*α*_s_ peptide binding to A_2A_R, constructed based on the crystallographic complex of active A_2A_ receptor and an engineered G protein (PDB: 5G53). **c** Histogram of de-convoluted components from ^19^F NMR spectra of the labeled G*α*_s_ peptide.


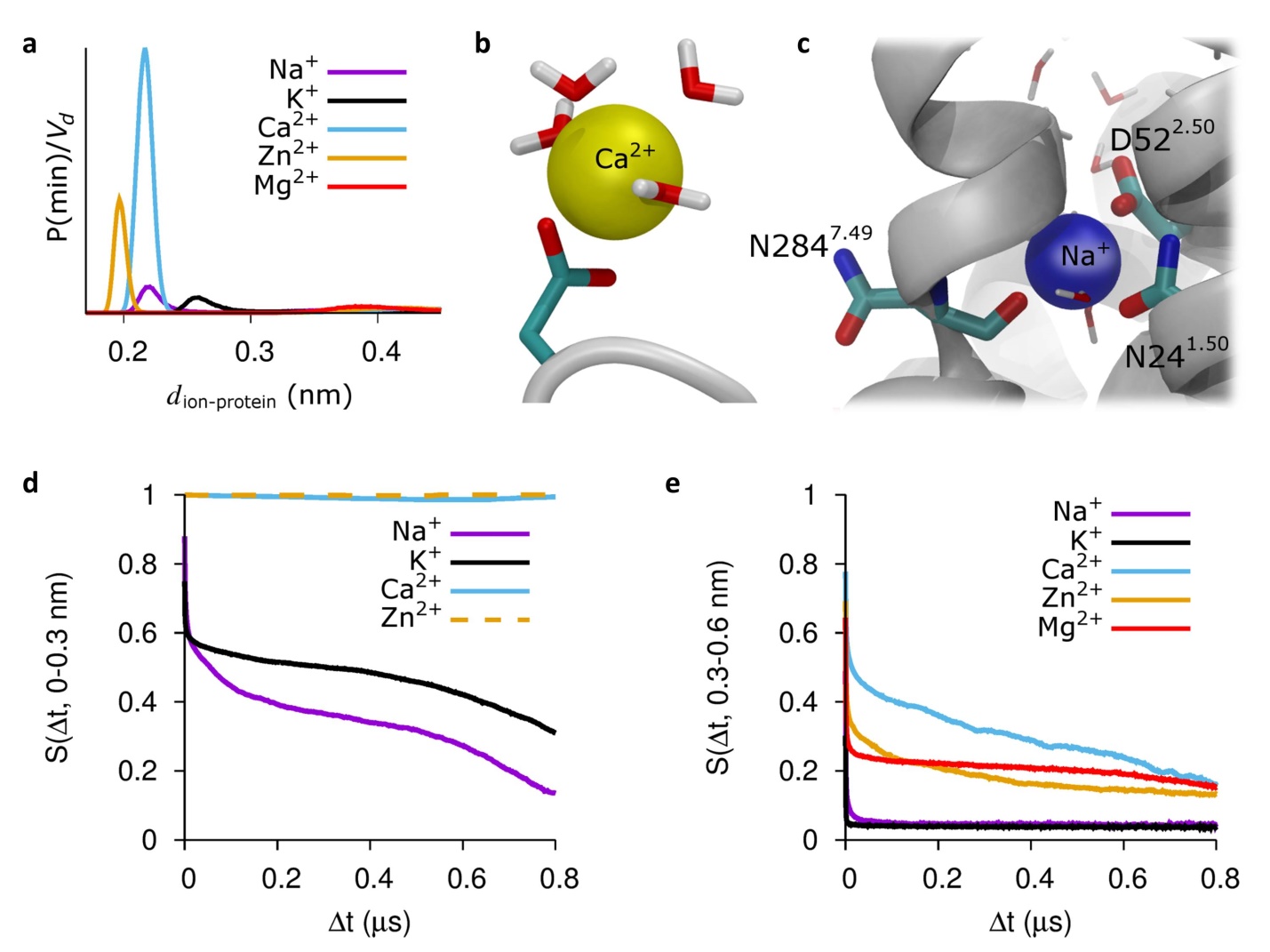
Supplementary Figure 4. Ionic desolvation and specificity of protein-cation interactions. a Histograms showing the minimum distance between each ion and any receptor atom, *d*_ion-protein_, normalized by radial volume. b Desolvation of a Ca^2+^ ion against an aspartate ion. c Desolvation of a Na^+^ ion against N24^1.50^, D52^2.50^, and the backbone carbonyl of N284^7.49^ in the region of the Na^+^-binding-pocket defined by Liu *et al.* [^1^](#_ENREF_1) . d and e, Survival probabilities, *S*, of ion-protein interactions in the range (d) 0-0.3 nm and (e) 0.3-0.6 nm, where *S*(*∆t*) represents the probability of being in a given state at time *t*+*∆t* given that this state was occupied at time *t*. Mg^2+^ is excluded from part (d) because it never became desolvated in these simulations (see part a). Data are from 15 simulations of three agonist-bound A_2A_R crystal structures [PDB IDs:2YDO, 3QAK and 5G53].


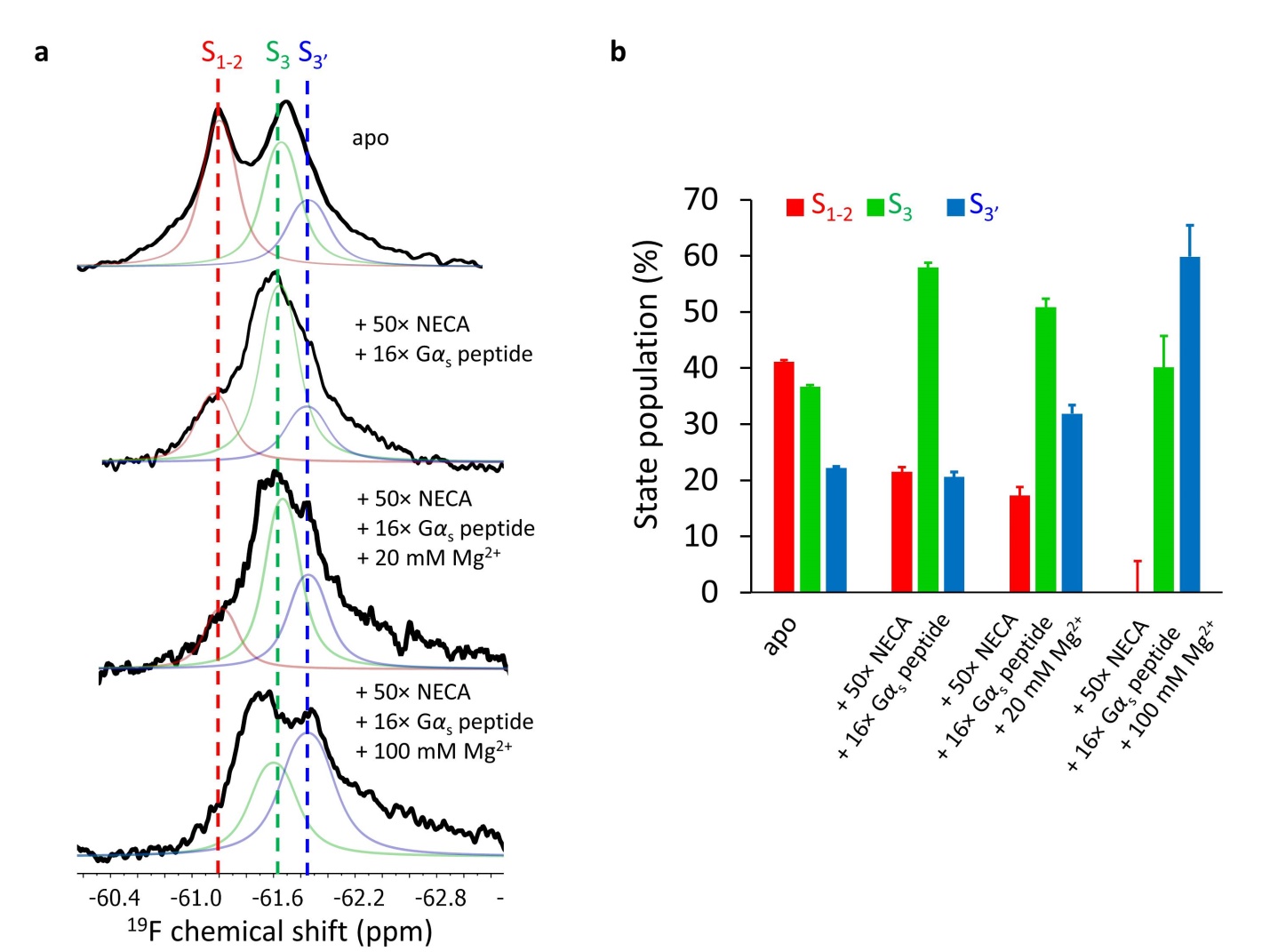


Supplementary Figure 5. Effects of Mg^2+^ on functional states of A_2A_R, in presences of NECA and G*α*_s_ peptide . All samples contain 100 mM NaCl. a ^19^F NMR spectra of 50 μM apo BTFMA-labeled A_2A_R-V229C as a function of Mg^2+^ concentration (0, 20 mM and 100 mM MgCl_2_), in presences of NECA and G*α*_s_ peptide. b Histogram of states, S_1-2_, S_3_ and S_3’_, recapitulated from the figure a.


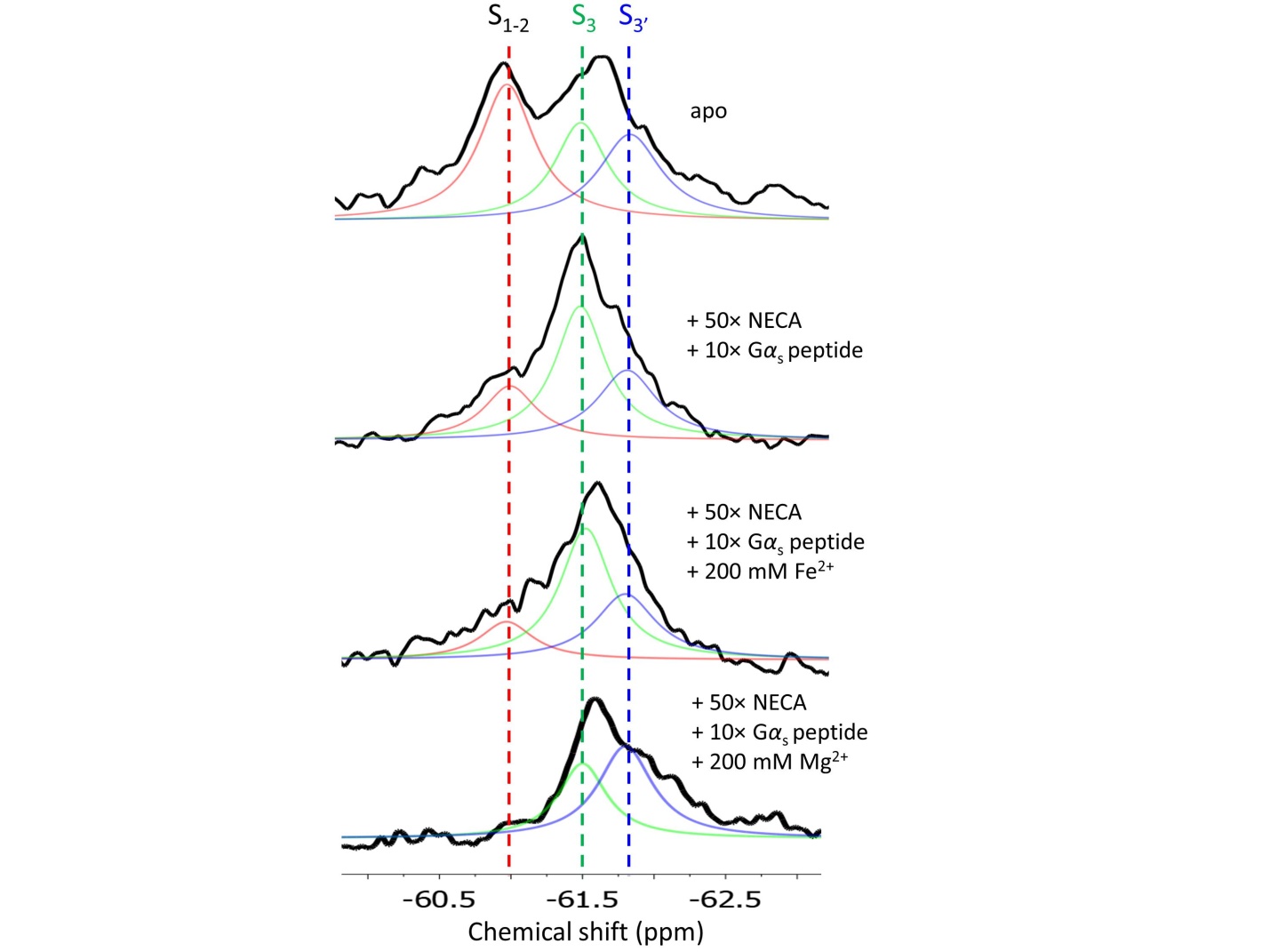


Supplementary Figure 6. Effects of agonist, Gα_s_ peptide, and divalent cations on A_2A_R conformational equilibria. ^19^F NMR spectra and deconvolutions into the S_1-2_, S_3_, and S_3’_ states. Note that the relative effects of Mg^2+^ are more pronounced than Fe^2+^ as shown in the bottom two spectra.


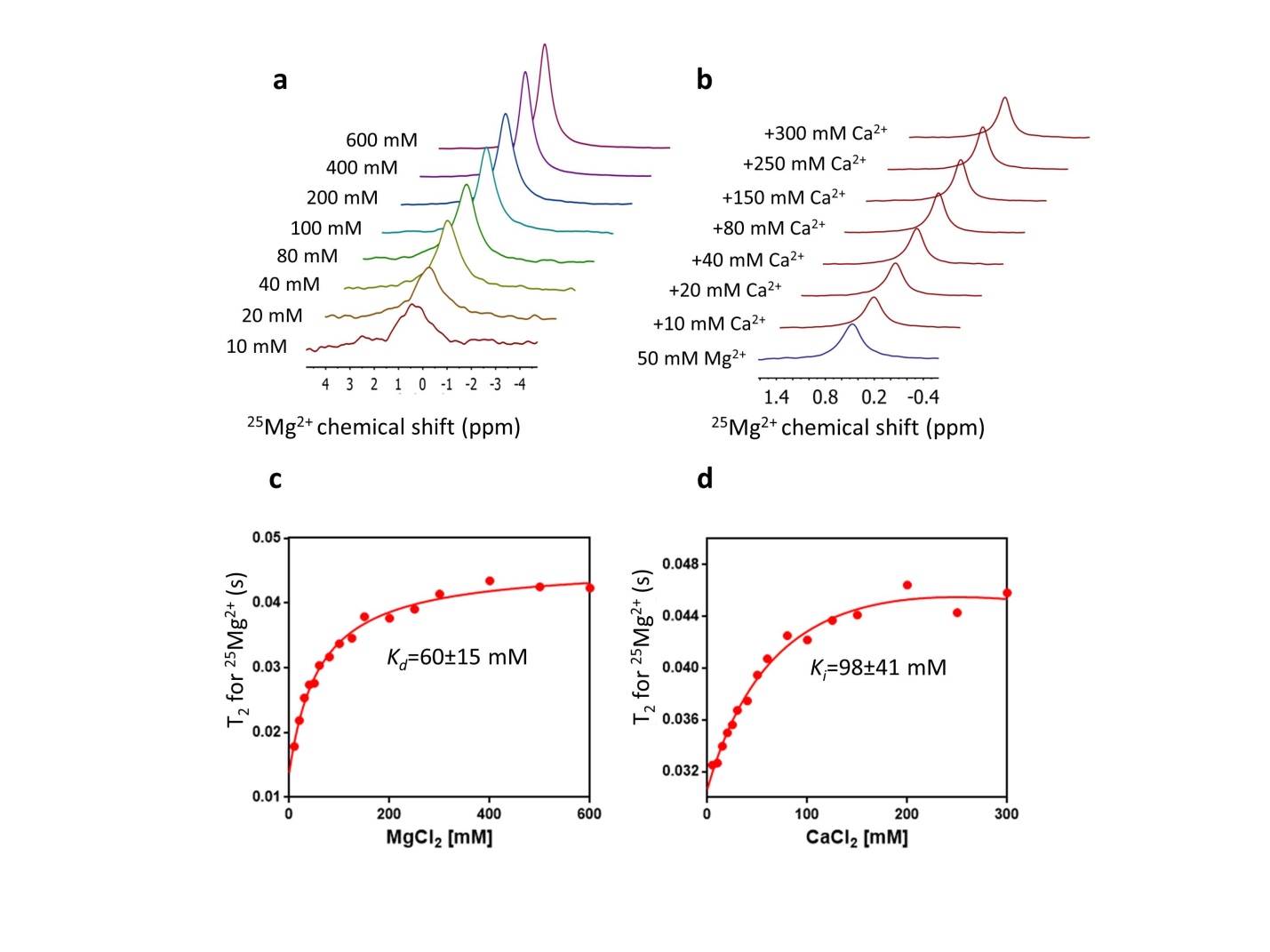


Supplementary Figure 7. Affinities of divalent caions measured by ^25^Mg NMR spectroscopy. a ^25^Mg NMR line width as a function of Mg^2+^ concentration (10-600 mM MgCl_2_) in the presence of 50 μM apo-A_2A_R-V229C. b ^25^Mg NMR line width as a function of Ca^2+^ concentration (10-300 mM CaCl_2_ and 50 mM MgCl_2_) in the presence of 50 μM apo-A_2A_R-V229C. c Line width based binding isotherm for MgCl_2_ derived from a, showing a dissociation constant of *K_d_*, 60 ±15 mM, obtained in the presence of 100 mM Na^+^. Note that the uncertainty is a result of a single experiment (N=1) and represents a fitting error. d Line width based binding isotherm derived from b, associated with 50 mM ^25^Mg^2+^ as a function of increasing CaCl_2_, showing a dissociation constant, *K_i_* , of 98±41 mM.

**Supplementary Table 1 Stable interactions between divalent cations and the A_2A_R in fifteen 1-μs simulations**

| Interaction^a^ | No. simulations observed | | | Figure |
| --- | --- | --- | --- | --- |
|  | 2YDO | 3QAK | 5G53 |  |
| D170-D261 bridging | - | 1 | - | 5B |
| E151-E161 bridging | 2 | 3 | 5 | 5C |
| E151-E169-D170 bridging | 1 | - | - | 5D |
| E169-D170 bridging | - | 2 | 1 | 5E |
| E169-lipid | - | 2 | - | - |
| E212-lipid | 1 | - | - | 5G |
| E219-lipid | - | - | 2 | - |
| E228^6.30^-lipid | - | - | 1 | - |
| D261-lipid | 4 | 4 | 2 | - |
| E312-lipid | 5 | - | - | - |
| E228^6.30^ direct binding | 1 | - | 1 | 5H |
| Na^+^-binding-pocket residence (<0.6 nm to D52^2.50^ or S91^3.39^)^b, c^ | 2 | 0 | 1 | 5F |
| Residence between the orthosteric pocket and the Na^+^-binding-pocket (<0.6 nm to E12^1.39^)^c^ | 4 | 1 | 3 | 5F |

^a^Interaction defined when noted binding partners are all closer than 0.3 nm to the divalent cation, except as noted.

^b^Sodium binding pocket defined by Liu *et al.*[_ENREF_17](#_ENREF_17)[^1^](#_ENREF_1).

^c^Interactions defined with the longer cutoff of *d*<0.6 nm are not generally stable on the μs timescale.

**Supplementary Reference**

1 Liu, W. *et al.* Structural basis for allosteric regulation of GPCRs by sodium ions. *Science* **337**, 232-236, doi:10.1126/science.1219218 (2012).
